# 85. Risk Factors and Outcomes of Resistant CMV Infections in Hematopoietic Cell Transplant Recipients: a 7-Year Review

**DOI:** 10.1093/ofid/ofaf695.031

**Published:** 2026-01-11

**Authors:** Marilyne Daher, Jennifer Makhoul, Vielka Lopez, Tali Shafat, Amy Spallone, Terri Lynn Shigle, Joseph Sassine, Anthony Febres, Oscar Morado-Aramburo, Ella Ariza Heredia, Fareed Khawaja, Roy F Chemaly

**Affiliations:** Infectious Diseases, Infection Control, and Employee Health, Houston, Texas; University of Texas Health Science Center at Houston/MD Anderson Cancer Center, Houston, TX; The University of Texas MD Anderson Cancer Center, Houston, Texas; The University of Texas MD Anderson Cancer Center, Houston, Texas; University of Texas MD Anderson Cancer Center, Houston, Texas; The University of Texas MD Anderson Cancer Center, Houston, Texas; University of Oklahoma Health Sciences Center, Oklahoma City, OK; The University of Texas at San Antonio, Houston, Texas; Hospital Beneficiencia Española, San Luis Potosi, San Luis Potosi, Mexico; The University of Texas MD Anderson Cancer Center, Houston, Texas; The University of Texas MD Anderson Cancer Center, Houston, Texas; University of Texas MD Anderson Cancer Center, Houston, Texas

## Abstract

**Background:**

Resistant/refractory CMV infection (R/R CMVi) is associated with high rates of morbidity and mortality in allogeneic hematopoietic cell transplant (allo-HCT) recipients. Primary letermovir prophylaxis was shown to decrease rates of R/R CMVi and 24-week all-cause mortality. There is limited data on breakthrough CMVi while on letermovir and the prevalence of de novo resistance. Our aim is to describe our experience of resistant CMVi, with a focus on letermovir-resistant CMVi.

**Table 1:**
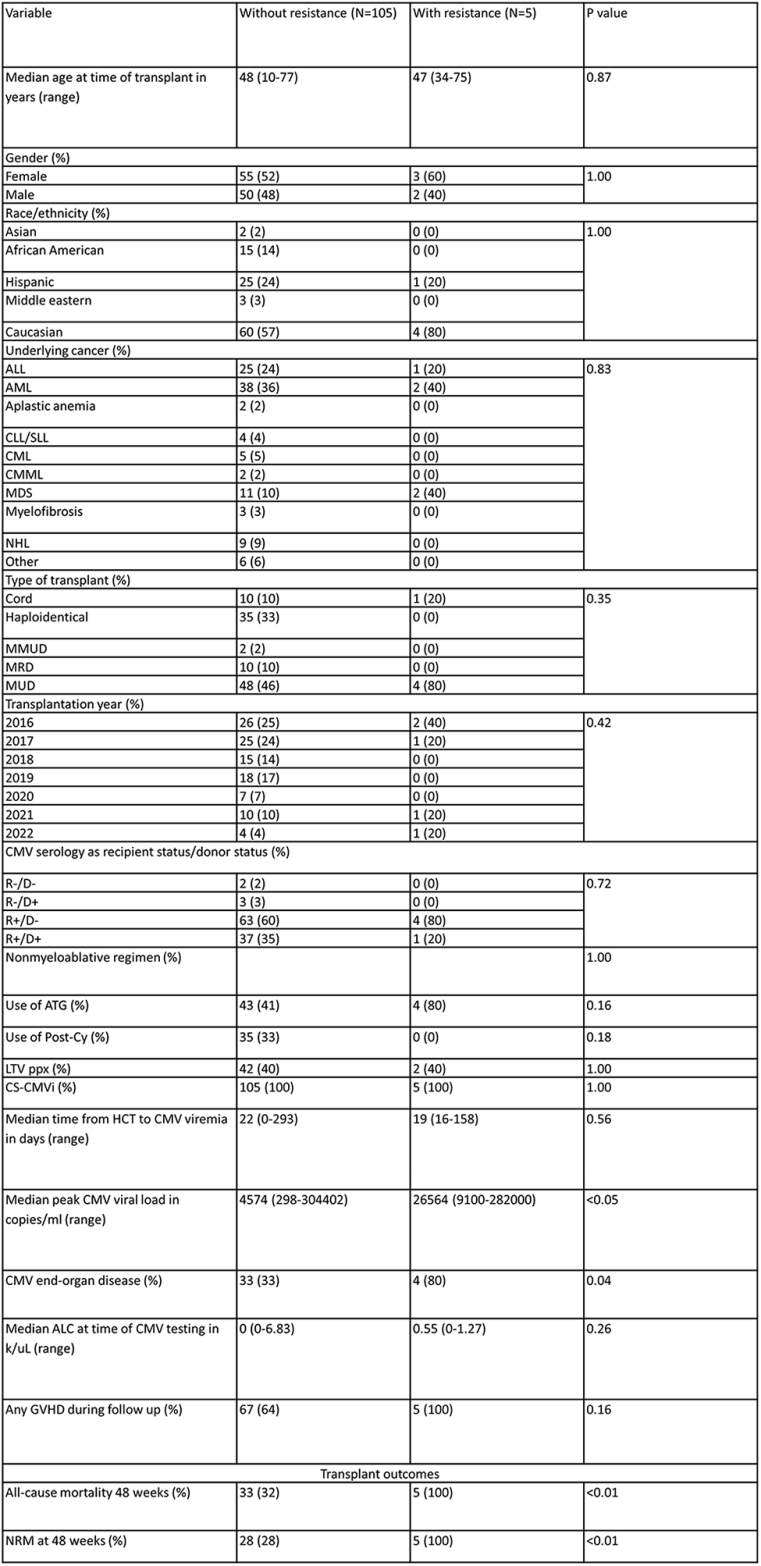
Comparison of Allo-HCT events with and without resistant CMV based on UL97 and UL54 analysis Abbreviations: Allo-HCT (allogeneic hematopoietic cell transplant); CMV, cytomegalovirus; ALL, acute lymphoblastic leukemia; AML, acute myeloid leukemia; CLL/SLL, chronic lymphocytic leukemia/small lymphocytic lymphoma; CML, chronic myeloid leukemia; CMML, chronic myelomonocytic leukemia; MDS, myelodysplastic syndrome; NHL, non-Hodgkin lymphoma; MMUD, mismatched unrelated donor; MRD, matched related donor; MUD, matched unrelated donor; R, recipient; D, donor; ATG, anti-thymocyte globulin; Post-Cy, post-transplant cyclophosphamide; LTV ppx, letermovir prophylaxis; CS-CMVi, clinically-significant CMV infection; ALC, absolute lymphocyte count; GVHD, graft-versus-host disease; NRM, non-relapse mortality

**Table 2:**
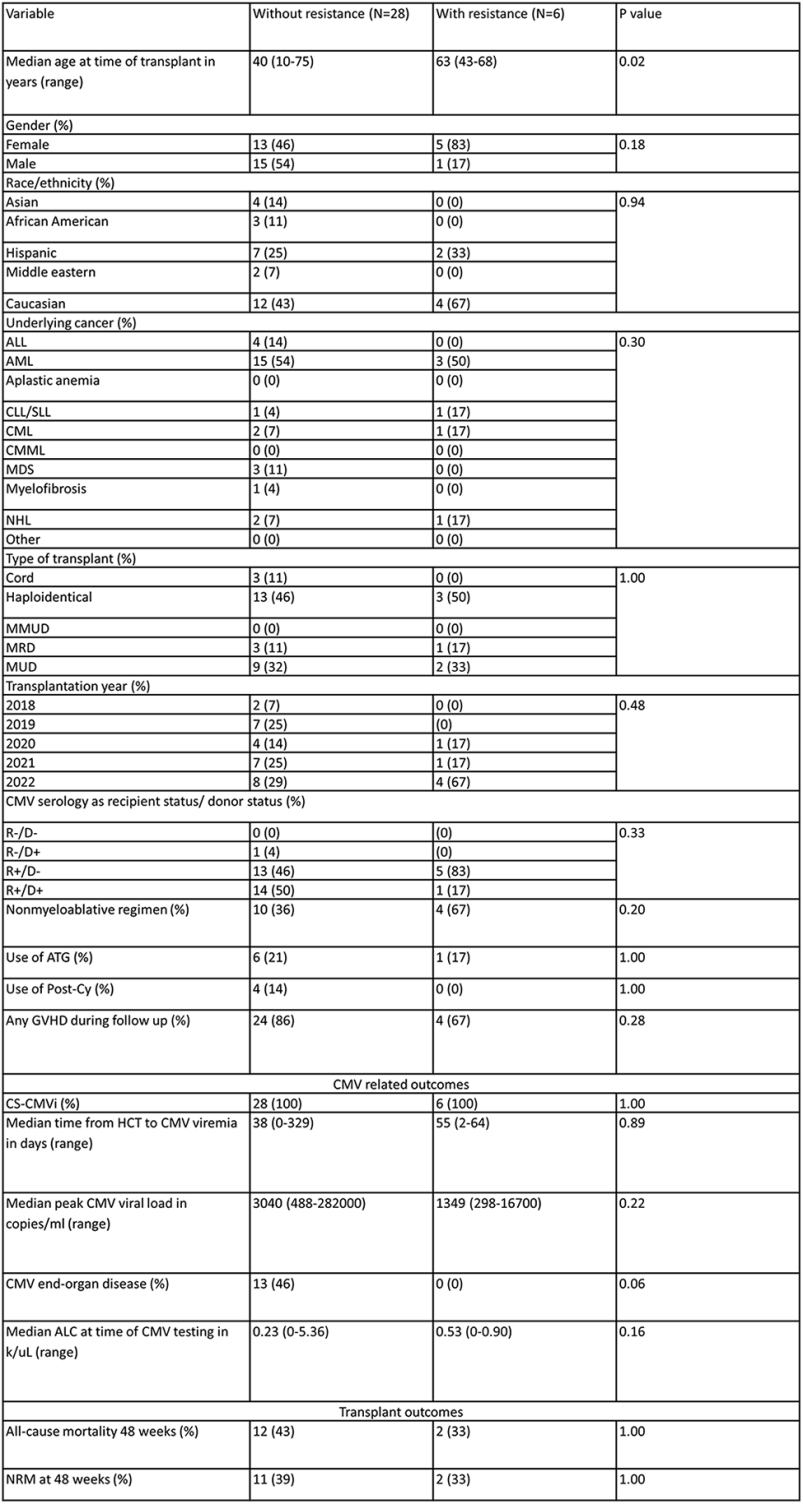
Comparison of Allo-HCT events with and without letermovir resistant CMV based on UL56 analysis Abbreviations: Allo-HCT (allogeneic hematopoietic cell transplant); CMV, cytomegalovirus; ALL, acute lymphoblastic leukemia; AML, acute myeloid leukemia; CLL/SLL, chronic lymphocytic leukemia/small lymphocytic lymphoma; CML, chronic myeloid leukemia; CMML, chronic myelomonocytic leukemia; MDS, myelodysplastic syndrome; NHL, non-Hodgkin lymphoma; MMUD, mismatched unrelated donor; MRD, matched related donor; MUD, matched unrelated donor; R, recipient; D, donor; ATG, anti-thymocyte globulin; Post-Cy, post-transplant cyclophosphamide; LTV ppx, letermovir prophylaxis; CS-CMVi, clinically-significant CMV infection; ALC, absolute lymphocyte count; GVHD, graft-versus-host disease; NRM, non-relapse mortality

**Methods:**

We reviewed all allo-HCT recipients who had CMV genotype testing for refractory or breakthrough CMVi at our institution between 2016 and 2023. We collected demographic, transplant-, CMV-, and cancer-related data. Two CMV genotype assays are available at our institution; one that tests for mutations at UL97 and UL54 that confer resistance to ganciclovir/maribavir and ganciclovir/cidofovir/foscarnet, respectively, and one that tests for UL56 mutations that confer resistance to letermovir. We performed univariate analysis using Fischer's exact test and Wilcoxon rank sum to compare risk factors and outcomes of patients with and without resistant CMVi.

**Table 3:**
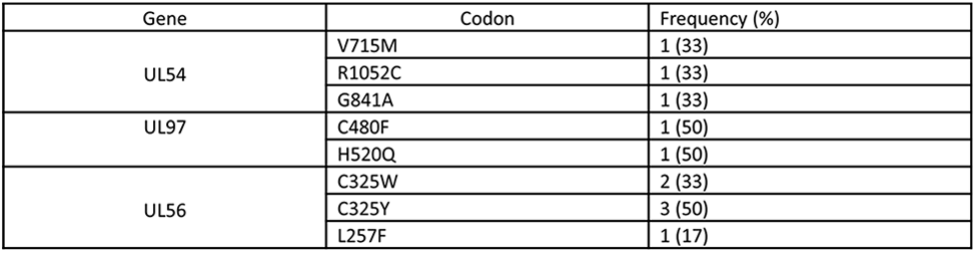
Frequency of CMV mutations conferring resistance

**Results:**

Of the 2571 HCT recipients, 110 had genotype testing for UL97 and/or UL54 and 34 for UL56. Of those, 2 (2%) and 3 (3%) had canonical mutations associated with resistance at UL97 and UL54, respectively, and 6 (18%) had mutations associated with resistance at UL56. Median peak CMV viral load and rate of CMV end-organ disease were higher in patients with UL97 or UL54 resistant CMVi (Table 1). Allo-HCT with letermovir breakthrough CMVi and UL56 mutations conferring resistance to letermovir were older at time of transplant (Table 2). All-cause and nonrelapse mortality at 48 weeks from transplant were higher in patients with UL97 and UL54 mutations but not for UL56. The specific mutations conferring resistance are shown in Table 3.

**Conclusion:**

Of 2571 HCT recipients, 5.6% had genotype testing, of which 7.6% had detectable resistance mutations, over half of which were associated with letermovir resistance. Older age was the only identifiable risk factor for UL56 resistance development. UL97 or UL54 mutations are associated with higher rate of CMV end-organ disease and mortality.

**Disclosures:**

Roy F. Chemaly, MD/MPH, ADMA Biologics, Inc.: Advisor/Consultant|AiCuris Anti-Infective Cures AG: Advisor/Consultant|AiCuris Anti-Infective Cures AG: Grant/Research Support|Ansun Biopharma Inc.: Advisor/Consultant|Ansun Biopharma Inc.: Grant/Research Support|Assembly Bioscience: Advisor/Consultant|Astellas Pharma Inc.: Advisor/Consultant|Eurofins-Viracor: Advisor/Consultant|Eurofins-Viracor: Grant/Research Support|Eurofins-Viracor: Honoraria|Gilead Biosciences: Advisor/Consultant|Invivyd, Inc.: Advisor/Consultant|Karius Inc.: Advisor/Consultant|Karius Inc.: Grant/Research Support|Merck and Company, Inc.: Advisor/Consultant|Merck and Company, Inc.: Grant/Research Support|Merck and Company, Inc.: Honoraria|Moderna, Inc.: Advisor/Consultant|Pfizer Pharmaceutc: Advisor/Consultant|Roche/Genentech: Grant/Research Support|SHIONOGI and CO., LTD.: Advisor/Consultant|Takeda Pharmaceutical: Advisor/Consultant|Takeda Pharmaceutical: Grant/Research Support|Tether: Advisor/Consultant

